# Multiple Insecticide Resistances in the Disease Vector *Culex p*. *Quinquefasciatus* from Western Indian Ocean 

**DOI:** 10.1371/journal.pone.0077855

**Published:** 2013-10-21

**Authors:** Nicolas Pocquet, Pascal Milesi, Patrick Makoundou, Sandra Unal, Betty Zumbo, Célestine Atyame, Frédéric Darriet, Jean-Sébastien Dehecq, Julien Thiria, Ambicadutt Bheecarry, Diana P. Iyaloo, Mylène Weill, Fabrice Chandre, Pierrick Labbé

**Affiliations:** 1 Institut de recherche pour le développement, Unité Mixte de Recherche MIVEGEC (IRD 224-CNRS 5290-UM1-UM2), Montpellier, France; 2 Université Montpellier 2, Institut des Sciences de l'Evolution de Montpellier (UMR 5554, CNRS-UM2-IRD), Montpellier, France; 3 Agence de Santé Océan Indien (ARS OI), St Denis, La Réunion Island, France; 4 DASS Nouvelle Calédonie, Santé Environnementale, Noumea, Territory of New Caledonia and Dependencies, Nouvelle Calédonie; 5 Vector Biology and Control Division, Ministry of Health and Quality of Life, Port Louis, Mauritius; University of Crete, Greece

## Abstract

Several mosquito-borne diseases affect the Western Indian Ocean islands. *Culex pipiens quinquefasciatus* is one of these vectors and transmits filariasis, Rift Valley and West Nile viruses and the Japanese encephalitis. To limit the impact of these diseases on public health, considerable vector control efforts have been implemented since the 50s, mainly through the use of neurotoxic insecticides belonging to Organochlorines (OC), Organophosphates (OP) and pyrethroids (PYR) families. However, mosquito control failures have been reported on site, and they were probably due to the selection of resistant individuals in response to insecticide exposure. In this study, we used different approaches to establish a first regional assessment of the levels and mechanisms of resistance to various insecticides. Bioassays were used to evaluate resistance to various insecticides, enzyme activity was measured to assess the presence of metabolic resistances through elevated detoxification, and molecular identification of known resistance alleles was investigated to determine the frequency of target-site mutations. These complementary approaches showed that resistance to the most used insecticides families (OC, OP and PYR) is widespread at a regional scale. However, the distribution of the different resistance genes is quite heterogeneous among the islands, some being found at high frequencies everywhere, others being frequent in some islands and absent in others. Moreover, two resistance alleles displayed clinal distributions in Mayotte and La Réunion, probably as a result of a heterogeneous selection due to local treatment practices. These widespread and diverse resistance mechanisms reduce the capacity of resistance management through classical strategies (e.g. insecticide rotation). In case of a disease outbreak, it could undermine the efforts of the vector control services, as only few compounds could be used. It thus becomes urgent to find alternatives to control populations of *Cx*. *p. quinquefasciatus* in the Indian Ocean.

## Introduction

**Figure 1:**
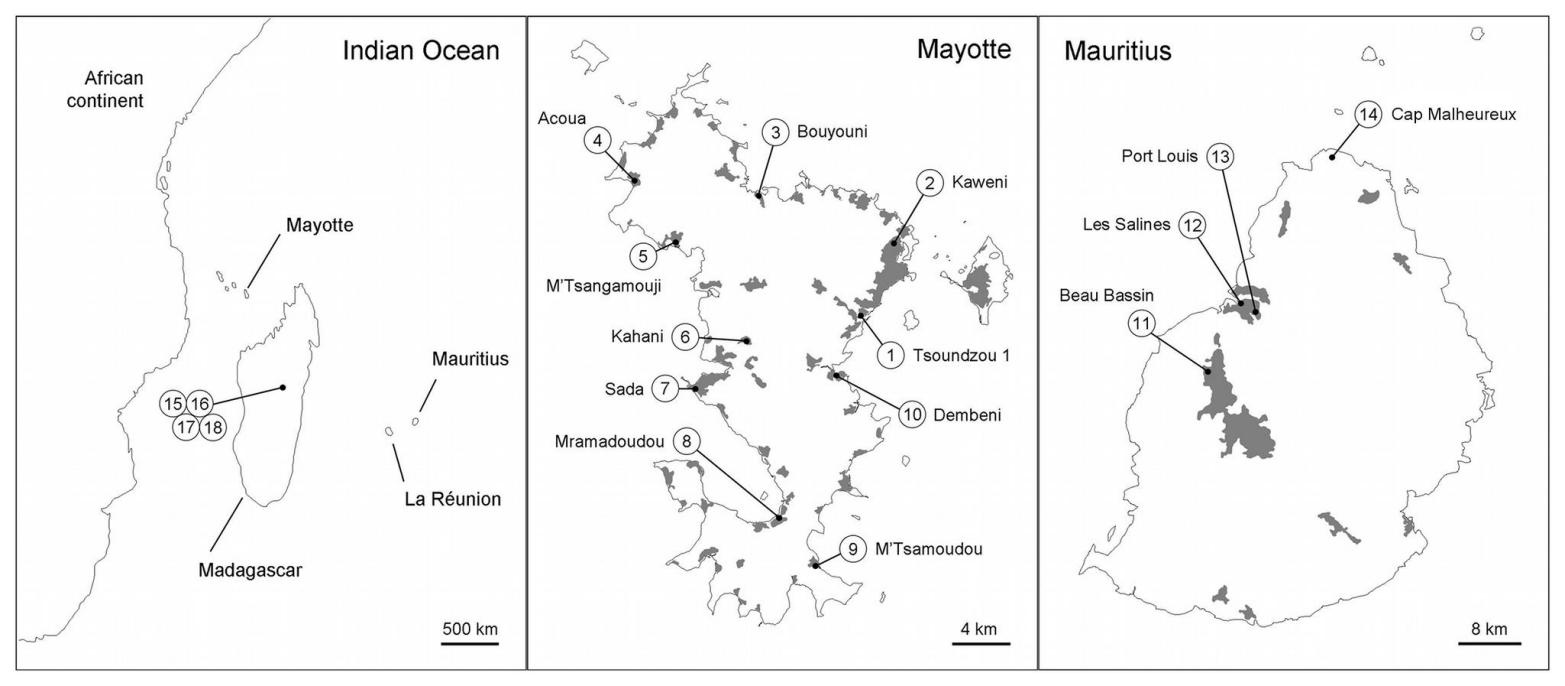
Sampled populations in the Indian Ocean. Samples from Mayotte are numbered from 1 to 10, samples from Mauritius are numbered from 11 to 14, and samples from Madagascar are numbered from 15 to 18. These numbers correspond to those of the samples in other tables and figures. The shaded areas correspond to urban areas.

Several vector-borne diseases, transmitted mainly by mosquitoes, have affected the Western Indian Ocean islands, i.e. the Comoros, the Mascarene Archipelago and Madagascar (Figure 1). The main ones are malaria transmitted by *Anopheles* species [1,2], dengue and chikungunya viruses transmitted by *Aedes* species [3-5]), and several filariasis transmitted by *Culex pipiens quinquefasciatus* [6]. This last species is also suspected to transmit the Rift Valley fever virus in the western part of the Indian Ocean [7-9] and is the vector of the West Nile virus and Japanese encephalitis at a worldwide scale [10]. Considerable efforts in vector control have therefore been carried out since the early 50s, in order to limit the impact of these diseases on public health [6,11]. 

In the western Indian Ocean islands, *Cx*. *p. quinquefasciatus* control was mainly implemented through the use of neurotoxic insecticides belonging to the Organochlorines (OC), the Organophosphates (OP) and the Pyrethroids (PYR) families [11-13]. The larvae of this species grow easily in breeding sites such as sewers or other wastewater collections[14], where in addition to insecticide treatments, they are also subject to a wide range of xenobiotics. In the field, mosquito control failures have been shown to result from resistant individuals, selected in response to insecticide exposure. However, the xenobiotics used for other purposes than mosquito control are present in *Cx*. *p. quinquefasciatus* breeding sites and may also have a role in the development of resistance, as suggested for other mosquito vector species [15-18]. Despite such repeated failures in mosquito control, very few data on insecticide resistance in *Cx*. *p. quinquefasciatus* are available for the Indian Ocean, with the recent exception of La Réunion Island [12]. 

The two main insecticide resistance mechanisms in mosquitoes are enzymatic detoxification (i.e. metabolic resistance) and target site modification (review in [19,20]). The major classes of enzymes involved in metabolic resistance are cytochrome P450 oxidases, esterases and glutathione-S-transferases. All classes are involved in resistance to different insecticides families, but oxidases play a major role in resistance to PYR, while esterases are mainly involved in resistance to OP. One esterase allele in particular, encoded by the *Ester* locus and named *Ester* ^2^, is found all over the world in *Cx. pipiens* populations (in both *pipiens* and *quinquefasciatus* subspecies, [21]). Another class of enzymes -the DDT-dehydrochlorinases (DDTases)- is particularly involved in resistance to DDT (DichloroDiphenylTrichloroethane, an OC). The second common resistance mechanism is the insecticide target modification. There are only few targets for neurotoxic insecticides, the main ones being the axonic voltage-gated sodium channels (Na-channels), the synaptic acetylcholinesterase (AChE1), encoded by the *ace-1* gene, and the synaptic γ-aminobutyric acid receptor (GABA receptor), encoded by the *Rdl* gene. These different target proteins are highly constrained, and their variability limited, since they play a key role in the nervous system [22]. In *Cx*. *p. quinquefasciatus*, the most common target modifications are the L1014F mutation (*kdr* ^*R*^ allele) in the voltage-gated sodium channel gene, conferring resistance to PYR and DDT, the G119S *ace-1* mutation (*ace-1* ^*R*^ allele), conferring resistance to OP and carbamates, and the A302S *Rdl* mutation (*Rdl* ^*R*^ allele), conferring resistance to the OC dieldrin [12,23,24].

In this study, the resistance levels of *Cx*. *p. quinquefasciatus* to the three main insecticide families used in vector control (PYR, OP and OC) were evaluated for the first time in two of the western Indian Ocean islands. Resistance mechanisms were characterized in samples from ten populations distributed throughout Mayotte Island, four populations from Central Madagascar and four populations from Mauritius. Among them, the frequencies of four major resistance alleles were more particularly assessed: *kdr* ^*R*^, *ace-1* ^*R*^, *Ester* ^2^ and *Rdl* ^*R*^.

Mayotte is a small mountainous island, with a majority of coastal roads except for the two roads crossing from east to west. The Northeast is the most densely populated area (Figure 1). Consequently this area is the main target for insecticide use. Thanks to a sampling scheme covering the totality of Mayotte, we were more specifically able to assess the impact of the heterogeneity of selective pressure and the role of migration on the distribution of insecticide resistance to various insecticides. 

Finally, comparisons between Mayotte, Madagascar, Mauritius and a previous study from La Réunion Island [12] allowed a first regional assessment of insecticide resistance in the major disease vector *Cx*. *p. quinquefasciatus* in the western Indian Ocean.

## Materials and Methods

### 2.1: Mosquitoes samples and strains

None of the samples in any location were collected in protected areas, and these field studies did not involve endangered or protected species. No specific permission was required to collect mosquito larvae in public areas, and when collected on private land or in private residences, the owners or residents gave permission for the study to be conducted on their land or in their residences.

*Cx*. *p. quinquefasciatus* larvae were collected in ten localities of Mayotte in 2011 (Figure 1), in various types of breeding sites (latrines, sewer…). Larvae were reared to adults in the laboratory and a sample was stored in liquid nitrogen for later analyses. For the Tsoundzou I sample (number 1 in Figure 1), some of the remaining adults were used in biochemical assays (see below) while the rest was bred to establish a laboratory strain (TZ1), and was thus maintained for several generations. Preliminary bioassays were carried out on the first generation (TZ1-F1) to identify the presence of any resistance in the field sample. This strain was then split in four replicates, each selected with a different insecticide to which TZ1-F1 showed resistance, in order to identify the responsible mechanism(s): TZ1-per, TZ1-tem and TZ1-chlor were respectively selected with permethrin (PYR), temephos (OP) and chlorpyrifos (OP) for six generations, and TZ1-diel was selected with dieldrin (OC) for seven generations.

Eight other samples were collected in 2010 from two other Indian Ocean islands, Madagascar and Mauritius (Figure 1; samples described in [25]). Adults were kept in liquid nitrogen for later analyses. One sample -collected at Les Salines in Mauritius (number 12 in Figure 1)- was maintained in the laboratory to establish a laboratory strain. The first generation (MAU-F1) was tested using preliminary bioassays to identify the presence of any resistance in the field sample. This strain was then split in two replicates, each selected with a different insecticide to which MAU-F1 showed some resistance, in order to identify the responsible mechanism(s): MAU-per and MAU-chlor were thus respectively selected with permethrin (PYR) during eight generations (MAU-per) and with chlorpyrifos (OP) during nine generations (MAU-chlor).

Finally, two laboratory strains were used in this study. The strain Slab [26] was used as the susceptible reference strain. Slab is susceptible to all the insecticides tested in this study. The second strain, SGaba, shared the same genetic background as Slab but homozygous for the *Rdl* ^*R*^ allele. This strain was established through eleven backcrosses of 200 females from Montpellier area (France) and carrying the *Rdl* ^*R*^ allele on males from the Slab strain; at each generation, the progeny were selected using 0.025 ppm of dieldrin to kill the susceptible homozygotes. After these backcrosses, the individuals carrying *Rdl* ^*R*^ were allowed to mate for three generations, their progeny being selected as above. Crosses between *Rdl* ^*R*^ homozygotes allowed obtaining the SGaba strain. 

### 2.2: Bioassays

 Larval bioassays were performed as described by Raymond et al. [27], using ethanol solutions of permethrin (PYR), DDT (OC), temephos (OP), chlorpyrifos (OP) and dieldrin (OC) (all compounds were purchased from Dr Ehrenstorfer GmbH, Germany). They were conducted on sets of 20 early 4^th^-instar larvae placed in a cup with 99 ml of water. One milliliter of the tested insecticide solution was then added in each cup. Assays of four to thirteen doses in a minimum of two replicates per dose were performed for each insecticide. Similar tests were performed in presence of different synergists: (i) the 1,1-bis-(p-chlorophenyl) methyl carbinol (DMC, Dr Ehrenstorfer GmbH, Germany), a DDT dehydrochlorinases inhibitor (DDTases, [28,29]), (ii) the piperonyl butoxide (PBO, Dr Ehrenstorfer GmbH, Germany), an inhibitor of some P450 oxidases [30], and (iii) the S,S,S-tributyl-phosphorotrithioate (DEF, Dr Ehrenstorfer GmbH, Germany), an inhibitor of some esterases and some GST [31]. Larvae were exposed to classical sublethal doses of one synergist 4 hours before adding the insecticide (DMC: 2 mg.L^-1^, PBO: 5 mg.L^-1^, DEF: 0.08 g.L^-1^). In all assays, larval mortality was recorded after 24 hours of insecticide exposure.

Mortality data were analyzed using the Priprobit software [32] based on Finney [33]. It allows testing the linearity of dose-mortality response and computing its slope and standard deviation. It also calculates the dose of insecticide necessary to kill 50 % of the tested sample (Lethal Concentration 50, or LC_50_) and the associated confidence intervals. Finally, it allows the comparison of two dose-mortality lines and the resistance ratios calculation, or RR (= LC_50_ of field sample / LC_50_ of the reference strain) and the synergism ratios, or SR (= LC_50_ in absence of synergist / LC_50_ in presence of synergist) and their 95 % confidence interval.

### 2.3: Metabolic resistance

Biochemical tests were performed on single 2-5 days-old females reared from 1^st^-instar larvae from the TZ1 sample to evaluate the activity of the main families of detoxification enzymes. Protein activity was quantified in microplates using the method of Bradford [34], the quantity or activity of the different detoxifying enzymes being expressed per mg of protein present in the homogenate or quantity of molecules metabolized per minute, respectively. Cytochrome P450 monooxygenases (sometimes named Mixed Function Oxidases or MFO) were quantified indirectly by the peroxidase activity of the heme group with tetramethylbenzidine (note that all hemoproteins are thus quantified, not only MFO [35]), esterases by their ability to hydrolyze α-naphthyl and β-naphthyl acetates and GST by their ability to conjugate reduced glutathione and chlorodinitrobenzene [36].

Statistical comparisons of detoxification enzyme activity present in mosquitoes of the susceptible strain Slab and of the TZ1 sample were computed using Mann-Whitney tests with the Statistica software [37]. 

Over-produced esterases (*Ester* locus) were investigated in Mayotte samples using starch gel electrophoresis, according to Pasteur et al. [38]; thorax homogenates were used. Esterase activity was revealed using α- and β-naphthyl acetates (as substrates) and Fast Garnett salts as dye. The esterases encoded by the different *Ester* alleles were identified by their electrophoretic mobility. For Mauritius and Madagascar samples, the *Ester* locus was studied by PCR as described by Berticat et al. [39], after total DNA extraction of single mosquitoes using a CTAB protocol [40].

Statistical analyses to compare the phenotypic frequencies at the *Ester* locus between samples were performed using the R software (http://www.r-project.org/) through a generalized linear model (GLM).

### 2.4: Analyses of target-site modifications

The frequencies of the various phenotypes associated to the presence/absence of susceptible/resistant acetylcholinesterase-1 (AChE1), encoded by the *ace-1* gene, were measured in Mayotte samples (except TZ1) using the TPP test described by Bourguet et al. [41]. For TZ1, Mauritius and Madagascar samples, the G119S mutation was investigated using the PCR-RFLP test described by Weill et al. [42], after total DNA extraction of single mosquitoes (CTAB protocol [40]). Both techniques provide the same information on the mosquito phenotypes ([RS] for heterozygotes, and [SS] or [RR] for susceptible and resistance allele homozygotes, respectively), so that their results are identical for a given individual. The choice on which method was used depended on whether the samples were conserved in liquid nitrogen (allowing the rapid TPP test on proteins) or in alcohol (where only the slower PCR test was usable).

For all samples from the different islands, genotyping of *kdr* and *Rdl* mutations was performed using a molecular test. Total DNA of single mosquitoes was extracted using the CTAB protocol [40]. The L1014F substitution causing resistance in the *kdr* gene was identified using the PASA method described in Martinez-Torres et al. [23]. The A302S substitution causing resistance in the *Rdl* gene was detected using the PCR-RFLP test described by Tantely et al. [12]. 

The frequency data from the *ace-1*, *kdr* and *Rdl* genes were analyzed using the Genepop software [43]. Hardy-Weinberg equilibrium was checked for each sample. Genotypic differentiation of the different Mayotte samples was computed by comparing each pair of samples with each locus. A p-value correction was applied using the sequential Bonferroni method to take multiple testing into account [44].

## Results

### 3.1: High resistance levels and several resistance mechanisms were identified by bioassays and detoxification enzyme activities

Our first goal was to identify the different mechanisms of resistance present in Mayotte and in Mauritius. To this aim, we analyzed two strains through bioassays and biochemical assays. We used a strain derived from one sample collected in Tsoundzou I (Mayotte) and named TZ1, and another strain derived from a sample collected in Les Salines (Mauritius) and named MAU. 

#### 3.1.1: TZ1 strain from Mayotte

Bioassays carried out on the first generation of the TZ1 strain (TZ1-F1) revealed resistance to the four tested insecticides when compared to the susceptible reference strain Slab, i.e. permethrin (PYR), chlorpyrifos and temephos (OP) and dieldrin (OC). Most assays suggested that the TZ1-F1 contained a mixture of susceptible and resistant individuals for the different insecticides tested (data not shown). 

This heterogeneity was further investigated by analyzing (a) TZ1 field mosquitoes for the genes coding the target proteins of pyrethroids (*kdr* gene), organophosphates (*ace-1* gene) and cyclodienes (*Rdl* gene) that can be identified by biochemical or molecular tests, (b) the global activity of different detoxifying enzymes (MFO, esterases and GST) on single mosquitoes of the TZ1-F1 strain, and (c) by re-analyzing dose-mortality responses in sub-strains derived from TZ1-F1 after six or seven generations of selection with permethrin (TZ1-per), temephos (TZ1-tem), chlorpyrifos (TZ1-chlor) and dieldrin (TZ1-diel). 

**Figure 2:**
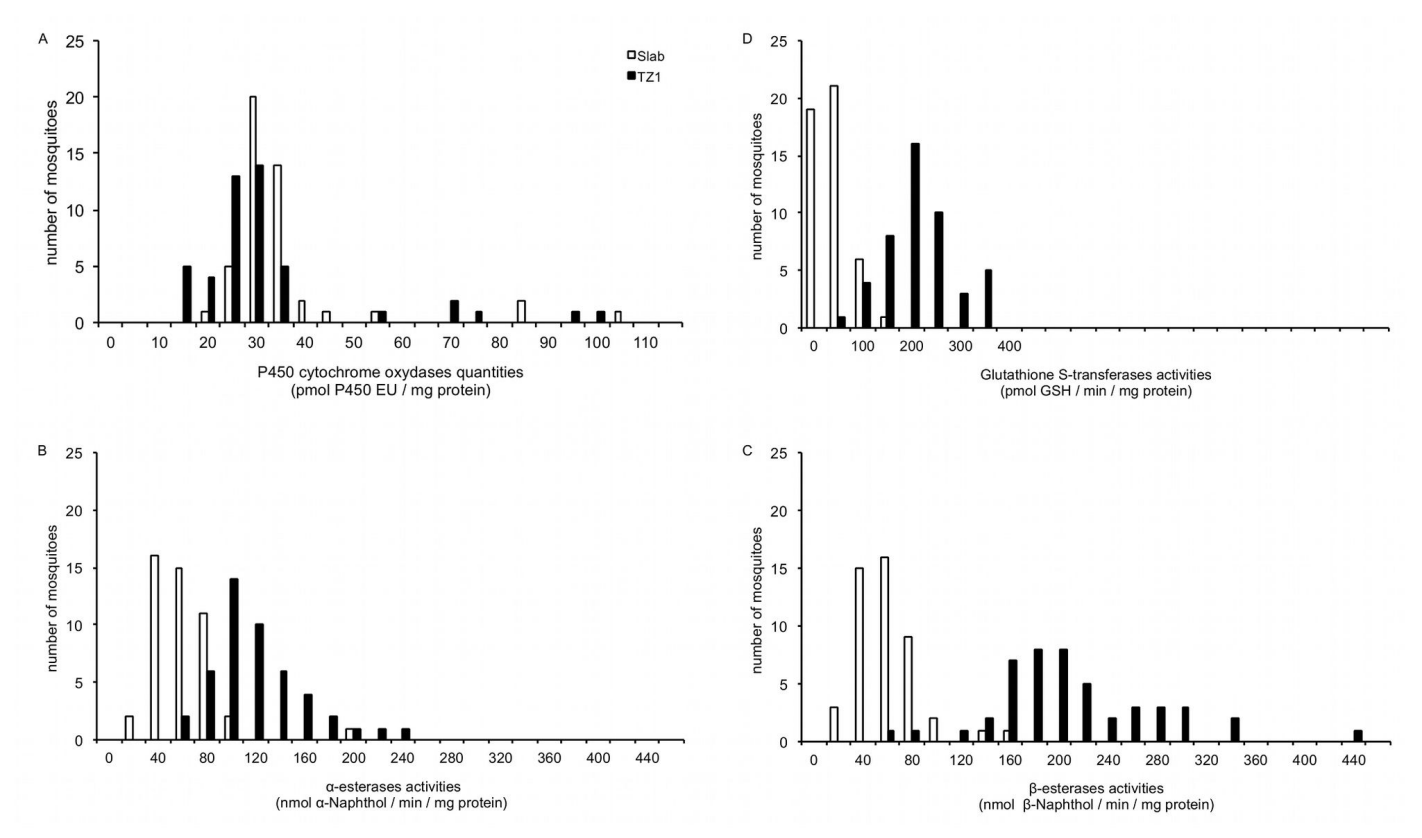
Comparison of detoxification enzymes quantities or activities in single mosquitoes of Slab and TZ1. A: The amount of cytochrome P450 oxidase is expressed in pmol of P450 Equivalent Unit per mg of protein for each mosquito. B and C: Activities of α and β-esterases are expressed as nmol of product formed (α or β-naphthol) per minute and per milligram of protein. D: GST activities are expressed in pmol of product formed per minute per milligram of protein.

All analyzed mosquitoes from the TZ1 sample were homozygous for the *kdr* ^*R*^ and the *Rdl* ^*R*^ alleles (N = 35 and 34, respectively), and 31 individuals out of 35 carried the *ace-1* ^*R*^ allele (either homozygous or heterozygous). The distributions of esterases and GST global activity among individuals from TZ1 were significantly shifted towards higher values (p < 0.00005) compared to the distributions for Slab mosquitoes (Figure 2). In contrast the global quantity of MFO was slightly lower for TZ1 than Slab (p = 0.002).

After six generations of permethrin selection of a replicate of TZ1-F1 (TZ1-per) resistance to this insecticide reached a resistance ratio (RR) of 199, compatible with previous studies [23]. Permethrin bioassays conducted with PBO did not show significant synergy effect (p > 0.05) between TZ1-per and Slab, suggesting that increased MFO detoxification was not involved in the observed permethrin resistance, in good agreement with the observed low global activity of MFO. In addition, TZ1-per displayed a strong cross-resistance to DDT (RR = 804; Table 1 and Figure S1B in supporting information), which was not synergized by DMC (a DDT-dehydrochlorinase inhibitor; Table 2), and which was thus probably due to the *kdr* mutation. These results indicated that permethrin resistance in TZ1 strains was probably mostly due to the presence of the resistant allele of the Na-channel gene (*kdr* ^*R*^), and that other resistance mechanisms (if present) had probably a very low frequency and a minor role.

**Table 1:** Resistance levels of TZ1 and MAU strains.

Insecticide	Strain	Linearity	LC_50_ (95% CI)	Slope (SD)	RR (95% CI)	SR (95% CI)
Permethrin	Slab	**p = 0.02**	1.0 x 10^-3^ (9.4 x 10^-4^ - 1.1 x 10^-3^)	6.21 (0.36)	-	-
	TZ1-per	**p = 0.03**	1.8 x 10^-1^ (1.2 x 10^-1^ - 2.5 x 10^-1^)	2.01 (0.27)	199 (193 - 204)	-
	MAU-per	**p < 10^-2^**	5.7 x 10^-1^ (3.8 x 10^-1^ - 8.9 x 10^-1^)	1.37 (0.16)	641 (546 - 754)	-
Permethrin + PBO	Slab	**p < 10^-2^**	3.4 x 10^-4^ (2.4 x 10^-4^ - 4.5 x 10^-4^)	3.80 (0.60)	-	2.8 (2.4 - 3.1)
	TZ1-per	p = 0.34	4.9 x 10^-2^ (4.3 x 10^-2^ - 5.6 x 10^-2^)	3.42 (0.36)	145 (119 - 178)	4.0 (3.1 - 5.0)
	MAU-per	p = 0.58	4.3 x 10^-2^ (3.4 x 10^-2^ - 5.2 x 10^-2^)	2.18 (0.23)	135 (107 - 171)	15 (11 - 23)
DDT	Slab	p = 0.68	7.1 x 10^-3^ (6.6 x 10^-3^ - 7.7 x 10^-3^)	6.68 (0.84)	-	-
	TZ1-per	p = 0.64	5.5 x 10° (4.7 x 10° - 6.4 x 10°)	3.20 (0.46)	804 (687 - 939)	-
	MAU-per	p = 0.28	3.9 x 10° (3.1 x 10° - 4.8 x 10°)	2.17 (0.23)	605 (486 - 748)	-
DDT + DMC	Slab	p = 0.07	1.8 x 10^-2^ (1.5 x 10^-2^ - 2.1 x 10^-2^)	3.88 (0.40)	-	0.4 (0.3 - 0.5)
	TZ1-per	p = 0.31	1.1 x 10^1^ (9.1 x 10° - 1.3 x 10^1^)	2.13 (0.17)	615 (482 - 792)	0.5 (0.4 - 0.6)
	MAU-per	p = 0.25	2.6 x 10° (2.0 x 10° - 3.4 x 10°)	1.24 (0.11)	187 (131 - 270)	1.2 (0.8 - 1.6)
Temephos	Slab	p = 0.78	1.2 x 10^-3^ (1.1 x 10^-3^ - 1.2 x 10^-3^)	7.95 (0.45)	-	-
	TZ1-tem	p = 0.81	1.1 x 10^-1^ (9.5 x 10^-2^ - 1.3 x 10^-1^)	5.47 (0.83)	86 (83 - 89)	-
Temephos + DEF	Slab	p = 0.06	7.0 x 10^-5^ (5.7 x 10^-5^ - 9.5 x 10^-5^)	2.07 (0.36)	-	19 (17 - 21)
	TZ1-tem	p = 0.74	1.2 x 10^-2^ (1.0 x 10^-2^ - 1.4 x 10^-2^)	3.63 (0.38)	193 (156 - 240)	9.9 (8.3 - 12)
Chlorpyrifos	Slab	p = 0.80	4.6 x 10^-4^ (4.5 x 10^-4^ - 4.8 x 10^-4^)	8.90 (0.46)	-	-
	TZ1-chlor	p = 0.24	3.9 x 10° (3.0 x 10° - 5.1 x 10°)	1.57 (0.16)	8070 (6949 - 9381)	-
	MAU-chlor	p = 0.13	3.5 x 10° (2.2 x 10° - 6.6 x 10°)	0.69 (0.11)	6024 (4870 - 7558)	-
Chlorpyrifos + DEF	Slab	**p = 0.01**	8.5 x 10^-7^ (3.9 x 10^-7^ - 1.5 x 10^-6^)	1.07 (0.14)	-	275 (217 - 349)
	TZ1-chlor	p = 0.18	3.5 x 10° (2.8 x 10° - 4.3 x 10°)	1.73 (0.18)	3.7 x 10^6^ (2.3 x 10^6^ - 6.5 x 10^6^)	1.1 (0.8 - 1.5)
Dieldrin	Slab	p = 0.17	1.1 x 10^-3^ (9.7 x 10^-4^ - 1.2 x 10^-3^)	3.87 (0.30)	-	-
	TZ1-diel	p = 0.79	5.3 x 10^-1^ (4.8 x 10^-1^ - 5.8 x 10^-1^)	5.59 (0.74)	493 (419 - 574)	-

The resistance levels of TZ1 and MAU strains selected with permethrin, temephos, chlorpyrifos and dieldrin and the effect of synergist on these resistance levels are presented. p is the probability of linearity rejection (bold when significant), LC_50_ is expressed in mg/l, SD is the standard deviation associated with the slope, RR is the resistant ratio, SR (LC_50_ observed in absence of synergist/LC_50_ observed in presence of synergist) is the synergism ratio and CI indicates the confidence intervals associated.

**Table 2:** Frequencies of *ace-1* ^R^, *kdr* ^*R*^ and *Rdl* ^*R*^ alleles in the Indian Ocean islands.

n°	Origin	Samples	*ace-1* locus					*kdr* locus					*Rdl* locus			
			N	*ace-1^R^*	F_is_	p		N	*kdr^R^*	F_is_	p		N	*Rdl^R^*	F_is_	p
1	Mayotte	Tsoundzou I	35	0.61	-0.13	0.34		35	1.00	-	-		34	1.00	-	-
2	Mayotte	Kaweni	47	0.61	-0.10	0.34		23	0.98	-	-		46	0.75	0.49	**0.002**
3	Mayotte	Bouyouni	52	0.41	0.10	0.84		57	1.00	-	-		58	0.42	-0.16	0.29
4	Mayotte	Acoua	58	0.32	-0.14	0.22		25	1.00	-	-		24	0.10	0.35	0.21
5	Mayotte	M'Tsangamouji	58	0.26	-0.34	**0.006**		48	1.00	-	-		56	0.16	0.21	0.13
6	Mayotte	Kahani	49	0.22	-0.28	**0.048**		56	0.99	-	-		58	0.28	0.46	**<0.001**
7	Mayotte	Sada	55	0.15	-0.16	0.28		20	1.00	-	-		23	0.26	0.12	0.61
8	Mayotte	Mramadoudou	54	0.38	-0.53	**<0.001**		0	-	-	-		0	-	-	-
9	Mayotte	M'Tsamoudou	57	0.61	0.02	0.66		50	1.00	-	-		57	0.10	0.30	0.07
10	Mayotte	Dembeni	57	0.46	-0.37	**0.005**		57	0.90	0.10	0.41		58	0.44	0.34	**0.015**
		*Total*	*522*	*0.39*	*-0.08*	*0.08*		*371*	*0.98*	*0.14*	*0.10*		*414*	*0.38*	*0.47*	***<0.001***
11	Mauritius	Beau Bassin	48	0	-	-		44	0.05	-0.02	1.00		43	0	-	-
12	Mauritius	Les Salines	23	0	-	-		24	0.35	-0.22	0.38		24	0	-	-
13	Mauritius	Port Louis	24	0	-	-		22	0.39	-0.04	1.00		24	0	-	-
14	Mauritius	Cap Malheureux	24	0	-	-		22	0.05	-0.16	0.66		24	0	-	-
		*Total*	*119*	*0*	-	-		*112*	*0.18*	*0.03*	*0.75*		*115*	*0*	-	-
15	Madagascar	Antananarivo 1	21	0	-	-		18	0.42	-0.23	0.62		22	0	-	-
16	Madagascar	Antananarivo 2	20	0	-	-		19	0.68	0.05	1.00		22	0.02	-	-
17	Madagascar	Itaosy 1	24	0	-	-		19	0.47	-0.03	1.00		24	0	-	-
18	Madagascar	Itaosy 2	24	0.02	-	-		18	0.44	-0.10	1.00		19	0	-	-
		*Total*	*89*	*0.006*	-	-		*74*	*0.51*	*-0.05*	*0.81*		*87*	*0.006*	-	-

The frequency of the resistant alleles for the *ace-1*, *kdr* and *Rdl* locus are presented for field samples of *Cx*. *p. quinquefasciatus* from Mayotte (samples 1 to 10), from Mauritius (samples 11 to 14) and from Madagascar (samples 15 to 18). F_is_ indicates deficit (F_is_ > 0) or excess (F_is_ < 0) of heterozygotes for each sample. p is the probability that observations deviate from the Hardy-Weinberg expectations (bold when significant) and N is the number of tested mosquitoes. *NB*: for *ace-1*, the frequencies have been computed as if only single copy alleles were present (see text).

Two subsets of TZ1-F1 were selected with temephos (TZ1-tem) and chlorpyrifos (TZ1-chlor) during 6 generations. In TZ1-tem, temephos resistance reached a relatively high level (RR = 86), and this resistance was synergized by DEF; however the DEF synergism ratio (SR) of Slab was higher than that of TZ1-tem (19 versus 9.9), and the temephos resistance observed in TZ1-tem could not be attributed to the increased esterase detoxification (Table 1 and Figure S1E in supporting information). In a manner similar to TZ1-chlor, chlorpyrifos resistance was particularly high (RR = 8070) but the addition of DEF yielded no effect (SR = 1.1 vs. SR = 275 for Slab; Table 1 and Figure S1F in supporting information), suggesting that detoxifying esterases were not involved in the observed resistance. These results were unexpected considering the high esterase activity observed in the TZ1 field sample with α- and β-naphthyl acetates, two substrates known to be hydrolyzed by overproduced esterases involved in OP resistance [36]. It must be noted that *ace-1* ^*R*^, which confers a <10-fold resistance to temephos and a ~20-fold resistance to chlorpyrifos, was probably also selected in the TZ1-tem and TZ1-chlor substrains; this resistance is not synergized by DEF. However, detoxifying esterases and *ace-1* ^*R*^ when associated in a same mosquito provide a resistance that is mostly additive [45]: the extremely high chlorpyrifos resistance recorded in the TZ1-chlor strain is thus particularly difficult to explain. Studies on a Tunisian strain [46] reported a ~10,000 fold resistance to chlorpyrifos that, as in TZ1-chlor, was not synergized by DEF. 

Finally, a subset of TZ1-F1 was selected with dieldrin (TZ1-diel) during seven generations; resistance reached a high level (RR = 493 (419-574); Table 1). There is presently no known mechanism of dieldrin detoxification. To assess the level of resistance in a strain homozygous for the *Rdl* ^*R*^ allele of the synaptic GABA in absence of other resistance genes, we established the SGaba strain, a strain carrying this allele and sharing the same genetic background as Slab. Bioassays were conducted on this strain with dieldrin: while Slab LC_50_ was 9.5 x10^-4^ (6.8 x10^-4^ - 12.6 x10^-4^), SGaba LC_50_ reached 0.25 (0.21-0.30), thus RR = 264 (166-437), which is coherent to a previous study of the *Rdl* mutation (RR = 196, 176-216; [47]). This is not very different from the resistance level displayed by TZ1-diel, so that it is reasonable to assume that *Rdl* ^*R*^, which was fixed in this sample (N = 34, Table 2), explains most of this resistance.

#### 3.1.2: MAU strain from Mauritius

Bioassays conducted on the first generation of the MAU strain (MAU-F1) showed moderate resistance to chlorpyrifos (OP) and dieldrin (OC) and a large resistance to permethrin (PYR). In the MAU field sample, no *ace-1* ^*R*^ and *Rdl* ^*R*^ allele was observed (N = 23 and 24, respectively) and *kdr* ^*R*^ was found to be present in 8 of the 24 mosquitoes analyzed. The detoxifying enzyme activity was not studied in the MAU-F1 strain before it was split in substrains for selection with the different insecticides.

Resistance to permethrin reached a high level (RR = 641) in the MAU strain selected with permethrin for eight generations (MAU-per), compatible with previous studies [23]. As in Mayotte, MAU-per also presented a strong cross-resistance to DDT (RR = 605; Table 1), which was not synergized by the dehydrochlorinase synergist DMC. In addition, permethrin bioassays in presence of PBO synergist showed a significantly greater synergy in MAU-per than in Slab (SR = 15 and 2.8 respectively; Table 1 and Figure S1C in supporting information), indicating an increased detoxification by MFO in MAU-per. Thus, in Mauritius permethrin resistance involves both *kdr* ^*R*^ and MFO. 

Even if only a low tolerance to chlorpyrifos was found in the MAU-F1 strain (RR = 5.2), selection with chlorpyrifos for nine generations has resulted in a sharp increase in resistance (RR = 6024 in MAU-chlor). Tests with synergists were not performed on this strain. 

### 3.2: High heterogeneity of resistance genes in the Indian Ocean islands is revealed by biochemical and/or molecular identification tests

 The polymorphism and distribution of four resistance genes were investigated, i.e. the three genes encoding target proteins (*kdr*, *ace-1* and *Rdl*, Table 2) and a gene encoding detoxifying esterases (*Ester*, Table 3), in samples collected from 10 populations in Mayotte, 4 populations in Mauritius, and 4 other populations in Madagascar (Figure 1). Three of these four genes were also studied in La Réunion [12]. 

**Table 3:** Frequencies of [*Ester^0^*] and [*Ester^**2**^*] phenotypes in the Indian Ocean islands.

n°	Origin	Samples	Esterase phenotypes		
			N	[*Ester* ^*0*^]	[*Ester* ^*2*^]
1	Mayotte	Tsoundzou I	0	-	-
2	Mayotte	Kaweni	53	0.53	0.47
3	Mayotte	Bouyouni	56	0.43	0.57
4	Mayotte	Acoua	58	0.28	0.72
5	Mayotte	M'Tsangamouji	58	0.48	0.52
6	Mayotte	Kahani	58	0.33	0.67
7	Mayotte	Sada	58	0.66	0.34
8	Mayotte	Mramadoudou	54	0.44	0.56
9	Mayotte	M'Tsamoudou	58	0.19	0.81
10	Mayotte	Dembeni	58	0.36	0.64
		*Total*	*511*	*0.41*	*0.59*
11	Mauritius	Beau Bassin	48	0.38	0.63
12	Mauritius	Les Salines	24	0.17	0.83
13	Mauritius	Port Louis	24	0.13	0.88
14	Mauritius	Cap Malheureux	24	0.17	0.83
		*Total*	*120*	*0.24*	*0.76*
15	Madagascar	Antananarivo 1	18	0	1.00
16	Madagascar	Antananarivo 2	21	0.14	0.86
17	Madagascar	Itaosy 1	19	0	1.00
18	Madagascar	Itaosy 2	16	0	1.00
		*Total*	*74*	*0.04*	*0.96*

*Ester* phenotype frequencies are presented for 10 samples of *Cx*. *p. quinquefasciatus* of Mayotte (samples 1 to 10), 4 samples from Mauritius (samples 11 to 14) and 4 samples from Madagascar (samples 15 to 18).

The *kdr* ^*R*^ mutation, identified using a PCR test, was observed in all the field samples of Mayotte where its frequency was high, ranging from 0.90 to 1 (mean frequency = 0.98 island-wide). In Mauritius, the resistance allele *kdr* ^*R*^ was present in all samples and had frequencies ranging from 0.05 to 0.39 (mean frequency = 0.18). Finally in Madagascar, the *kdr* ^*R*^ allele displayed frequencies ranging from 0.42 to 0.68 (mean frequency = 0.51). The distribution of *kdr* in La Réunion is unfortunately unknown, but bioassays and PCR-tests showed that it was present in the island [12]. The *kdr* ^*R*^ allele is thus widely distributed among the four islands where it can provide a strong resistance to PYR and DDT.

The *Rdl* ^*R*^ mutation, identified using a PCR test, showed a more restricted distribution than *kdr* ^*R*^. It was observed in the nine studied samples collected in Mayotte. It was fixed in the TZ1 field sample and had frequencies ranging from 0.10 to 0.75 in the other Mayotte samples (mean frequency = 0.38, Table 2). Three samples (numbers 2, 6 and 10) showed a significant deficit of heterozygotes (p < 0.05), which was probably due to a Wahlund effect [48], i.e. a mixture of distinct sub-populations with reduced gene flow. The situation is quite similar to the one in La Réunion [12], where the frequency of *Rdl* ^*R*^ ranged from 0.08 to 1 (mean frequency = 0.56). Finally, the *Rdl* ^*R*^ allele was not found in any of the four field samples collected in Mauritius and was observed in a single mosquito (heterozygous) among the four studied samples from Madagascar.

The polymorphism of the *ace-1* gene can be detected indifferently through a molecular PCR-RFLP test or through a biochemical assay, TPP. Both tests were used in this study. In Mayotte, the TZ1 field sample was analyzed with the PCR-RFLP test and *ace-1* ^*R*^ was found to have a frequency of 0.61. The nine other samples were investigated using the TPP test. The resistant allele *ace-1* ^*R*^ was present throughout the island, with frequencies ranging from 0.15 to 0.61 (mean frequency = 0.39, Table 2). Among the ten Mayotte samples, four showed significant deviations from Hardy-Weinberg expectations due to an excess of heterozygotes (p < 0.05). Over all samples there was a close-to-significant excess of heterozygotes (p = 0.08). Such excesses of heterozygotes suggested the presence of duplicated haplotypes combining a susceptible and a resistant copy of the *ace-1* gene (allele *ace-1* ^*D*^) [49-51]. Such haplotypes have been identified in three samples of Mayotte by crossing experiments (as described in [50]) and are currently being further analyzed (unpublished data). The *ace-1* locus was analyzed by PCR test in the other islands. In the four samples from Mauritius, all individuals showed a susceptible genotype for *ace-1*, suggesting that the resistant allele is absent from the island or present at a very low frequency (N = 119). In Madagascar only one heterozygous individual (sample 18) was found among the four tested samples. In La Réunion, the frequency of *ace-1* ^*R*^ ranged from 0 to 0.29 (mean frequency = 0.05) [12].

The *Ester* locus can also be analyzed indifferently by biochemical or molecular tests. Both tests only identify the presence or absence of a resistant *Ester* allele. In Mayotte only two phenotypes were found, [*Ester* ^*0*^] corresponding to a susceptible homozygote, and [*Ester* ^*2*^], corresponding to (*Ester2*/*Ester* ^*°*^) and (*Ester2*/*Ester* ^*2*^) genotypes. The [*Ester* ^*2*^] phenotype was found in all tested samples, with frequencies ranging from 0.34 to 0.81, and a mean frequency of 0.59. In Mauritius, the resistant phenotype [*Ester* ^*2*^] was found in the four samples, with frequencies ranging from 0.63 to 0.88 (mean frequency = 0.76, Table 3). In Madagascar, the [*Ester* ^*2*^] phenotype was found in the four studied samples at very high frequencies (from 0.86 to 1, mean frequency = 0.96). Finally in La Réunion, the frequency of the [*Ester* ^*2*^] ranged from 0 to 0.88 (mean frequency = 0.18) [12]. 

### 3.3: Spatial distribution of resistance genes in Mayotte

**Figure 3:**
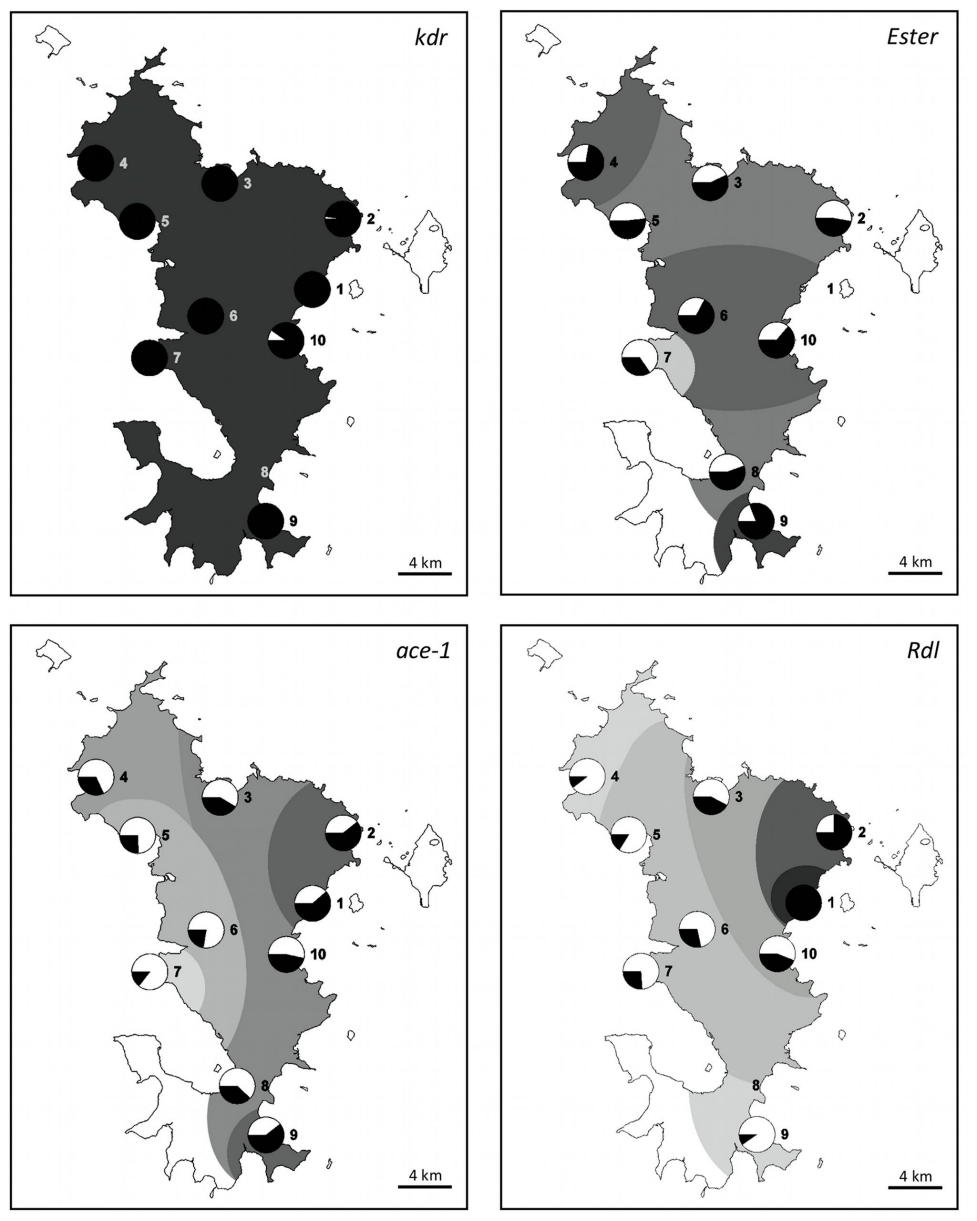
Geographic distribution of *kdr*, *Ester*, *ace-1* and *Rdl* resistant alleles in Mayotte. For each sample, the frequencies of resistant alleles (*kdr* ^*R*^, *ace-1* ^*R*^, *Rdl* ^*R*^) or phenotypes ([Ester^2^]) are represented in black sectors in a circle. The shaded areas approximately correspond to the statistical groups observed (see text), with a scaled shade of gray ranging from 0 (white) to 1 (black) corresponding to the mean frequency of the corresponding group.

Thanks to our sampling scheme all across the island, it was possible to analyze the distribution of the resistance alleles. Apart from the *kdr* ^*R*^ allele, which was close to fixation all over the island, the resistance alleles analyzed here displayed structured distributions (Figure 3).

 Analysis of the *Ester* ^2^ phenotype distribution did not show any particular pattern (Figure 3). Samples were statistically grouped according to their frequency as follows: two groups contained samples presenting no significant differences (samples 2, 3, 5 and 8, and samples 4, 6 and 10), although there was a significant difference between these two groups. Samples 7 and 9 were different from all other samples with, respectively, the lowest and the highest frequency of the island (0.34 and 0.81). However, these differences displayed no clinal pattern across the island.

 Concerning *ace-1*, the resistant allele displayed a strong and heterogeneous spatial pattern, the average *ace-1* ^*R*^ frequency decreased from east to west (Figure 3). Statistical analyses revealed four groups showing significant genotypic frequency differences. The first group, in the east of the island, was formed by samples 1, 2, 3, 9 and 10, with an average *ace-1* ^*R*^ frequency of 0.54. The second and the third groups were formed by samples 3, 4, 8, 10 and samples 3, 4, 5, 6 and 8 with respective average *ace-1* ^*R*^ frequencies of 0.39 and 0.32. The last group, formed by samples 5, 6, and 7, had an average *ace-1* ^*R*^ frequency of 0.21. The four groups partially overlapped (leading to five frequency classes, see Figure 3) and *ace-1* ^*R*^ frequency decreased as one moved away from Tsoundzou and Kaweni (samples 1 and 2, north-east) or from M'Tsamoudou (sample 9, south-east).

The *Rdl* alleles also displayed a marked variation in their spatial distribution over Mayotte (Figure 3). The resistant allele *Rdl* ^*R*^ frequency seemed to decrease as one moved away from Tsoundzou (sample 1), as shown by the negative correlation between *Rdl* ^*R*^ frequency and the distance from there (Pearson correlation: r = -0.89, p = 0.001). *Rdl* ^*R*^ frequencies ranged from complete fixation in Tsoundzou I to 0.10 in samples 4 and 9, respectively the most eastern and the most southern collection sites on the island. Statistically homogeneous but overlapping groups emerged as follows: sample 1 (Tsoundzou I), sample 2 (close to the north of Tsoundzou I), samples 3, 6, 7 and 10, samples 4, 5, 6 and 7 and samples 4, 5, 7 and 9, with respective *Rdl* ^*R*^ average frequencies of 1, 0.75, 0.35, 0.20 and 0.16.

## Discussion

In the Indian Ocean, the mosquito *Cx*. *p. quinquefasciatus* is an important vector of several diseases, including filariasis, Rift Valley fever and West Nile viruses, and the Japanese encephalitis. In this study we investigated its status of resistance to the most commonly used insecticides to control its population densities (including the diseases it transmits). So far, there is no available data for the western Indian Ocean islands, except for La Réunion [12]. We investigated Madagascar and two of the main archipelagos -the Comoros, Mayotte and the Mascarenes (Mauritius)- in order to build the first regional assessment of insecticide resistance for this important vector.

### a): Resistance to a large variety of insecticides is widespread in the western Indian Ocean

In the western Indian Ocean, *Cx*. *p. quinquefasciatus* presents resistances and/or resistance mechanisms to all the main insecticide families used so far in vector control, i.e. PYR (permethrin), OP (chlorpyrifos, temephos) and OC (dieldrin, DDT). The most common resistance mechanism to PYR is the *kdr* ^*R*^ mutation, which also confers resistance to DDT (OC); it was found through the whole region. In La Réunion and Mauritius, metabolic resistance due to an increased MFO detoxification was also present, but it was not found in Mayotte. The level of resistance to PYR in Mayotte and Mauritius is high, as expected from the presence of *kdr* ^*R*^ in this species [23]. 

OP resistance through esterase overexpression, especially the *Ester* ^2^ allele, is widespread and found at high frequencies in all the sampled western Indian Ocean islands. The *ace-1* ^*R*^ mutation is also present in the area, although is less common (ex. not found in Mauritius). Our knowledge of the resistance to chlorpyrifos conferred by these two resistance genes does not explain the high resistance to chlorpyrifos (OP) observed after the selection of Mayotte and Mauritius field samples (TZ1 and MAU, > 6,000 folds after selection). Such an extremely high resistance to this insecticide has only been reported in Tunisia (> 10,000 folds; [46,52]) where it involved a new gene (named G) associated with resistant *ace-1* ^*R*^. It is possible that this gene is present in Mayotte and Mauritius, and possibly in other Indian Ocean islands, but further studies are needed to confirm it. Finally, dieldrin (OC) resistance through the *Rdl* ^*R*^ mutation has also been detected in some of the sampled islands, but not all. 

In conclusion, in this study we used different and complementary approaches to describe the variety of resistance mechanisms in the Indian Ocean islands (bioassays, measures of enzyme activities, molecular identification of target-site mutations). Clearly, resistance to the most-used insecticides families is widespread at a regional scale; however, the distribution of these resistance mechanisms is quite heterogeneous among the islands.

### b): Regional heterogeneity of resistance is probably due to vector control practices

There are indeed important differences among the western Indian Ocean islands for the frequencies of the different resistance mechanisms present. This is particularly the case for the *kdr* ^*R*^ allele: it is close to fixation in Mayotte (mean frequency = 0.98), but less frequent in Madagascar and Mauritius (0.51 and 0.18, respectively). In La Réunion, the *kdr* ^*R*^ frequency is unknown, but the allele was found in a strain selected with permethrin and DDT was used in the island for malaria control [12]. These differences are probably related to the insecticides used in vector control: in Mayotte, DDT was used from 1973 to 1984 [11], and then replaced by deltamethrin (PYR). This insecticide is still used for indoor residual spraying (IRS) and deltamethrin-treated nets have recently been distributed in the island (Zumbo, pers. com.). Forty years of such intense selection pressure on the sodium channel gene, the common target of PYR and DDT, explain the near-fixation of this allele in this island. The lower *kdr* ^*R*^ frequencies observed in Madagascar and Mauritius seems to indicate that the selection pressures on this gene, i.e. the intensities of PYR and DDT treatments, are certainly less important in these two islands. This seems surprising for Mauritius, as DDT has been continuously used from 1946 to 2011 for malaria control [13]. However, from 1990 on, the doses used could have been low enough to weaken the selection pressure intensity (ex. only 2 rounds per year of DDT spraying around the airport, [13]). The *kdr* ^*R*^ distribution could also be structured (in Mauritius, *kdr* ^*R*^ frequency ranged from 0.05 to 0.4, Table 2). Finally, *kdr* ^*R*^ may also have been selected in La Réunion and Mauritius by reinforced vector control of *Aedes* species following chikungunya and dengue outbreaks[12,53]. 

Metabolic resistance to PYR is also contrasted between the islands, as MFO implication in resistance has been detected in Mauritius and La Réunion [12], but not in Mayotte (no available data for Madagascar). Considering the intensity of PYR used in Mayotte, the fact that no MFO-based metabolic resistance has been detected is surprising. One plausible explanation is that at the time of this study temephos (OP) was still intensively used in Mayotte (see below): temephos is bio-activated to temephos-oxon (the toxic form) by oxidases [54]; if the same oxidases are implicated in both temephos activation and permethrin resistance, it might thus be possible that the intense use of temephos in Mayotte could have led to a counter-selection of oxidases. Further studies are required to establish this point. 

Heavy uses of OP insecticides have been documented in most of these islands: temephos was used for vector control in Mayotte from 1973 to the end of 2010 ([11]; Zumbo, pers. com.), until 2006 in La Réunion [12], and from 1975 to at least 2008 in Mauritius [13]; no information is available for Madagascar. In all four islands, *Ester* ^2^ is present at high frequencies, with some samples reaching frequencies of 0.8 in Mayotte, 0.9 in Mauritius and up to 1 in Madagascar and La Réunion (Table 3 and [12]). It suggests a relatively early spread of this resistance allele in the Indian Ocean, consistent with its highly invasive character [21,55], and appears a testimony of high OP selection pressure in all islands. However this selection may be due also to other OP and carbamates, for example those intended for agriculture and domestic usages. 

Consequently, the more contrasted distribution of *ace-1* appears surprising. The *ace-1* ^*R*^ allele is indeed only present in La Réunion and Mayotte (plus one heterozygote in Madagascar), and at much lower frequencies than *Ester* ^2^ (Table 2 and [12]). The absence of *ace-1* in Mauritius is particularly puzzling: temephos is indeed used since 1975, many exchanges occur between the different Indian Ocean islands; furthermore all susceptible individuals tested (data not shown) displayed a 119 codon allowing the G119S mutation in one step [56]. One potential explanation is that, as *ace-1* ^*R*^ provides low resistance to this OP (RR < 10, [27]), this limited advantage could, in certain treatment conditions, be unable to compensate its high fitness cost [57-61]. Another explanation for the discrepancies between *Ester* ^2^ and *ace-1* ^*R*^ frequencies could be that *Ester* ^2^ would be selected by some other products (ex. agriculture), not necessarily used in vector control, and for which *ace-1* is not the target. Esterases are indeed generalist detoxifying enzymes, able to provide protection against a large array of xenobiotics, including other insecticide families (ex. most PYR; [62,63]). Another observation is that the frequency of the *ace-1* resistance allele is very different between La Réunion and Mayotte: it is much lower in the first than in the second island (from 0 to 0.29, mean = 0.05, and from 0.15 to 0.61, mean = 0.39, respectively, Table 2 and [12]). This may be due to the presence of *ace-1* duplications in Mayotte, which were not found in La Réunion [12]. These duplicated alleles have been shown to provide resistance while reducing its fitness cost [50]. Although their frequencies still need to be evaluated, they may partly explain why *ace-1* resistance is more frequent in Mayotte. An alternative but not exclusive explanation could be that *ace-1* is currently invading the Indian Ocean from the northwest, i.e. recent importation from Eastern Africa or local mutation [12], which would explain why it is more frequent in Mayotte than in La Réunion, and so far absent or quasi–absent in Mauritius and Madagascar. Only long-term studies documenting the dynamics of the different resistance genes could help solve this issue.

Finally, the *Rdl* ^*R*^ allele conferring resistance to dieldrin exhibits a distribution very similar to that of *ace-1* ^*R*^: it is only found in Mayotte and La Réunion (and only one heterozygote in Madagascar) (Table 2 and [12]). Before being banned in France, dieldrin was the only insecticide targeting the GABA receptor used for vector control in these French overseas departments: it has been used in Mayotte from 1952 to 1958 [11], but never in La Réunion [12]. As the dieldrin half-life is 7 years in the soil [64], it is nevertheless unlikely that this legally-used dieldrin persisted in the environment to explain the current resistance. However, other insecticides, such as lindane and fipronil, target the GABA receptor [65] and are respectively used by veterinarians and against termites [12]. Traces of these compounds have been reported in La Réunion coastal waters, as well as traces of dieldrin, probably from illegal uses [12]. This could explain the selection of *Rdl* ^*R*^, although the presence of an unknown source cannot be excluded. 

### c): Local gradients in resistance frequency reveal heterogeneous insecticide pressure

At a local scale, the distribution of *kdr* ^*R*^, *Rdl* ^*R*^, *ace-1* ^*R*^ and *Ester* ^2^ were investigated in samples from 10 populations of *Cx*. *p. quinquefasciatus* throughout Mayotte. As a similar sampling scheme was performed in the previous study of La Réunion (except for *kdr*, [12]), we were able to compare the two islands and found that the distributions of resistance genes are particularly congruent.

Three of the genes present evidence of a strong structuration both in Mayotte and La Réunion, i.e. *Rdl* ^*R*^, *ace-1* ^*R*^ and *Ester* ^2^ (Figure 3 in the present study and Figure 2 in [12]). No clear spatial pattern emerged for *Ester* ^2^, either in La Réunion or in Mayotte (Figure 3): the gene was relatively frequent in both islands, with consequent variations between samples from different populations that probably reflect the heterogeneity of the selective agents in the environment. As discussed above, these selective agents may be the OP used in vector control, but also other xenobiotics, not used for vector control. Moreover, as *Ester* ^2^ can be relatively costly [57-61,66], the heterogeneity in its frequency distribution within an island could reflect a heterogeneity in the selective pressure intensity, i.e. the quantity of pesticide used.

Finally, both *Rdl* ^*R*^ and *ace-1* ^*R*^ showed gradient frequency distributions: in Mayotte both decreased from east to west, while they decreased from northwest to southeast in La Réunion; in both islands this gradient reflected the decreasing human population density gradient (Figure 3 in the present study and Figure 2 in [12]). While *ace-1* ^*R*^ has been repetitively shown to be quite costly in absence of OP [57-61], few data exist on the potential cost of *Rdl* ^*R*^ in absence of dieldrin, although it has been shown to usually decline in absence of the insecticide [67]. Their clinal distributions are thus probably the result of a more intense selection in the most populated areas associated with a decline due to their cost in less treated/less populated areas, with migration redistributing the different alleles [57]. Again, the source(s) of this selection is(are) not clearly identified for *Rdl* ^*R*^, while OPs or carbamates are the most probable cause for *ace-1* ^*R*^.

## Conclusion

The status of *Cx*. *p. quinquefasciatus* insecticide resistance in the Western Indian Ocean is particularly worrying. Indeed, resistance mechanisms to all the most commonly used neurotoxic insecticide families (PYR, OC and OP) are found over the entire region. Both site mutations (*kdr* ^*R*^, *Rdl* ^*R*^, *ace-1* ^*R*^) of their main targets (respectively, sodium channels, GABA receptor and AChE1) and metabolic resistance mechanisms (*Ester* ^2^, MFO) are present at a regional scale, sometimes close to fixation in the natural populations of this mosquito. Even more, a not yet identified mechanism providing extreme resistance to chlorpyrifos in mosquitoes carrying *ace-1* ^*R*^, and duplicated alleles of the locus *ace-1* are present. This type of multi-resistance is not uncommon and rather reflects the situation of many areas across the world for several mosquito species [68-75].

This resistance diversity gravely reduces the capacity of its management. Classical strategies indeed consist in insecticide family rotation, which would be very difficult at this stage considering the variety of mechanisms already present. It is even more worrying as such strategies rely on the existence of resistance fitness costs: unfortunately, resistances with reduced cost have already appeared (ex. *ace-1* duplications) and different resistance mechanisms can act in synergy (ex. the presence of *kdr* ^*R*^ largely limits the cost of *ace-1* ^*R*^; [61]). Moreover, large heterogeneities in the frequencies of the various resistance alleles were found, so that the control strategies should be precisely designed to adjust to the particular situation of each island.

In the case of *Cx*. *p. quinquefasciatus* in the Indian Ocean, the main risks are epidemics of Bancroftian filariasis [6,76,77] and the Rift Valley fever virus [7,9,78]. In case of an outbreak of either of these diseases, these already-established resistances could undermine the efforts of the vector control services. Temephos could still be used in emergency cases, but to do so the European legislation on this product should be changed, and the presence of resistance alleles could reduce its utility on a long-term basis.

In the meantime, alternative insecticides could also be potentially used to control an epidemic. Insect Growth Regulators (IGR) are efficient, but show very low persistence on *Cx*. *p. quinquefasciatus* at the currently used doses ([79,80]; Pocquet et al., unpublished data). *Bti* toxins (extracted from *Bacillus thuringiensis* var. *israelensis*) could be a serious alternative, however their residual efficiency is relatively short, particularly in tropical environments and polluted water ([81]; Pocquet et al., unpublished data).

Thus it becomes urgent to find alternatives to control populations of *Cx*. *p. quinquefasciatus* in the Indian Ocean. One of the most promising research paths is the development of Incompatible Insect Techniques (IIT). A first step in the development of these strategies has recently been performed: Atyame et al. [25] have introduced in a *Cx*. *p. quinquefasciatus* line a strain of *Wolbachia* incompatible with the strain present in the Indian Ocean. *Cx*. *p. quinquefasciatus* males of this new line could sterilize all females on most of the Indian Ocean islands. The development of such techniques would allow fighting effectively and specifically *Cx*. *p. quinquefasciatus* in this part of the world.

## Supporting Information

Figure S1 **Synergist effect on resistance levels of TZ1 and MAU strains selected to insecticides.** Each graph shows the dose-mortality of Slab and one selected strain for one insecticide, with or without synergist. Panel A: effect of permethrin on Slab and TZ1-per, with or without PBO. Panel B: effect of DDT on Slab and TZ1-per, with or without DMC. Panel C: effect of permethrin on Slab and MAU-per, with or without PBO. Panel D: effect of DDT on Slab and MAU-per, with or without DMC. Panel E: effect of temephos on Slab and TZ1-tem, with or without DEF. Panel F: effect of chlorpyrifos on Slab and TZ1-chlor, with or without DEF.
(TIF)
