# Supplementary figures and images for: Multiple Insecticide Resistances in the Disease Vector Culex p. Quinquefasciatus from Western Indian Ocean
Source: PLoS One. 2013 Oct 21;8(10):e77855. doi: 10.1371/journal.pone.0077855 (PMC3804603; doi:10.1371/journal.pone.0077855)

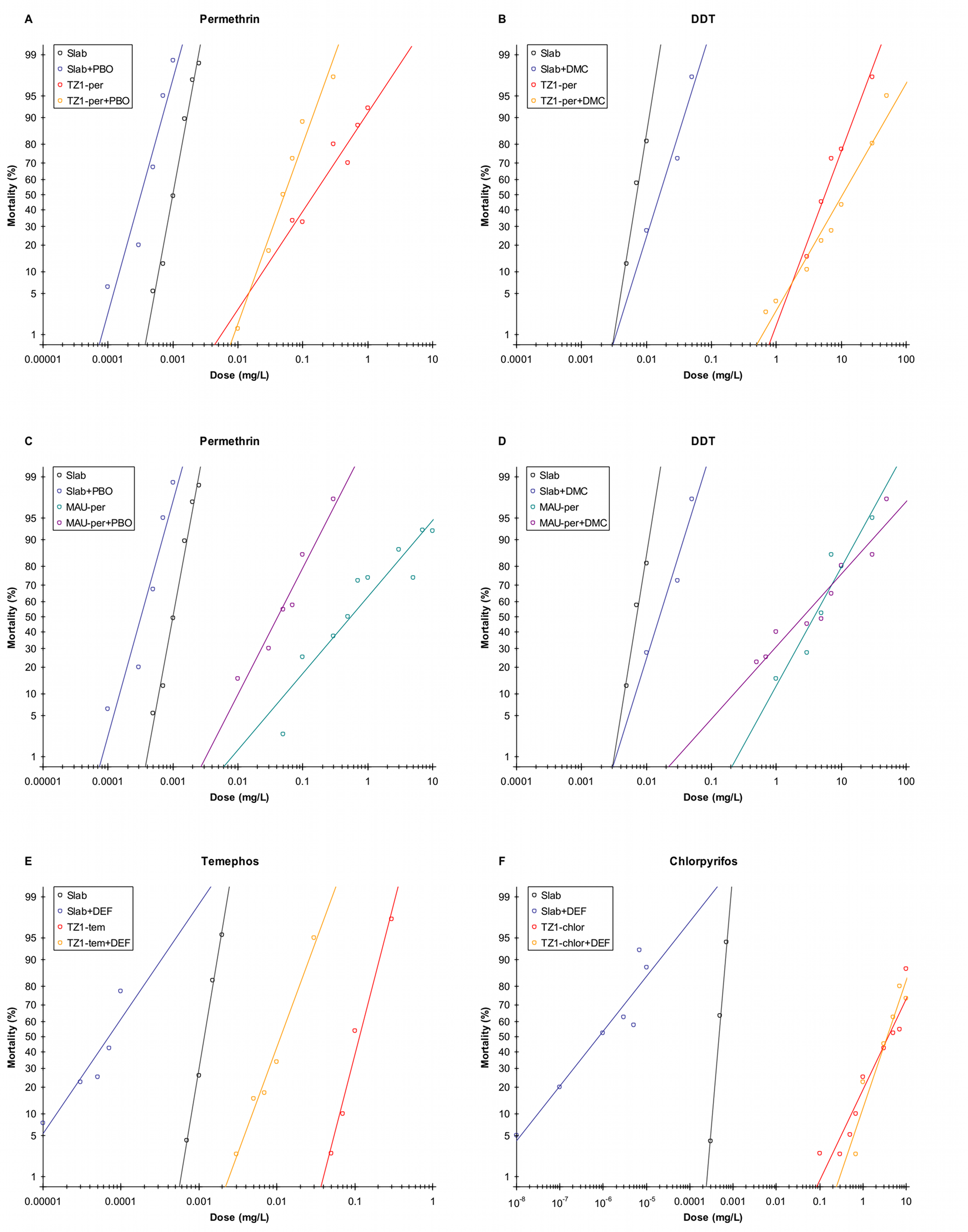

Supplement: Figure S1 — Synergist effect on resistance levels of TZ1 and MAU strains selected to insecticides. Each graph shows the dose-mortality of Slab and one selected strain for one insecticide, with or without synergist. Panel A: effect of permethrin on Slab and TZ1-per, with or without PBO. Panel B: effect of DDT on Slab and TZ1-per, with or without DMC. Panel C: effect of permethrin on Slab and MAU-per, with or without PBO. Panel D: effect of DDT on Slab and MAU-per, with or without DMC. Panel E: effect of temephos on Slab and TZ1-tem, with or without DEF. Panel F: effect of chlorpyrifos on Slab and TZ1-chlor, with or without DEF. (TIF) [file pone.0077855.s001.tif]
